# 

**Published:** 1961-10

**Authors:** 


### THE MEDICAL JOURNAL OF THE SOUTH-WEST

*Incorporating*
*The Bristol Medico-Chirurgical Journal*

*October 1961*

NATIONAL LIBRARY

JAN 28 1963

OF MEDICINE

VOL. 76 (iv). No. 282 Price 4/-

